# Supplementary material for: MDGA1 Gene Variants and Risk for Restless Legs Syndrome
Source: Int J Mol Sci. 2025 Jul 12;26(14):6702. doi: 10.3390/ijms26146702 (PMC12295006; doi:10.3390/ijms26146702)
Supplement: Supplementary file 1 [file ijms-26-06702-s001.zip › ijms-3722880-supplementary.pdf]

Supplementary Table S1. Genotypes and allelic variants of patients with RLS and healthy volunteers distributed by sex. The values in each cell represent number (percentage; 95% confidence intervals) P: crude probability; Pc: probability after multiple comparisons; NPV: negative predictive value.

**GENOTYPE**	***RLS WOMEN (N=204, 408 ALLELES)***	***CONTROL WOMEN (N=217, 434 ALLELES)***	***INTERGROUP COMPARISON OR (95%CI), P; PC ; NPV (95%CI)***	***RLS MEN (N=59, 118 ALLELES)***	***CONTROL MEN (N=63, 126 ALLELES)***	***INTERGROUP COMPARISON OR (95%CI) P; PC ; NPV (95%CI)***
rs10947690 A/A	138 (67.6; 61.2-74.1)	132 (60.8; 54.3-67.3)	1.35 (0.90-2.01); 0.145; 0.692; 0.56 (0.50-0.63)	40 (67.8; 55.9-79.7)	38 (60.3; 48.2-72.4)	1.36 (0.66-2.91); 0.392; 0.963; 0.57 (0.44-0.69)
rs10947690 A/G	60 (29.4; 23.2-35.7)	77 (35.5; 29.1-41.9)	0.76 (0.50-1.14); 0.184; 0.692; 0.49 (0.46-0.53)	17 (28.8; 17.3-40.4)	22 (34.9; 23.1-46.7)	0.75 (0.35-1.62); 0.472; 0.963; 0.49 (0.43-0.56)
rs10947690 G/G	6 (2.9; 0.6-5.3)	8 (3.7; 1.2-6.2)	0.79 (0.27-2.32); 0.670; 0.765; 0.51 (0.51-0.52)	2 (3.4; -1.2-8.0)	3 (4.8; 0.5-10.0)	0.70 (0.11-4.35); 0.704; 0.963; 0.51 (0.50-0.53)
rs61151079 C/C	174 (85.3; 80.4-90.2)	177 (81.6; 76.4-86.7)	1.13 (0.78-2.20); 0.305; 0.692; 0.57 (0.46-0.68)	50 (84.7; 75.6-93.9)	52 (82.5; 73.2-91.9)	1.18 (0.45-3.08); 0.743; 0.963; 0.55 (0.33-0.75)
rs61151079 C/CACGAGG	28 (13.7; 9.0-18.4)	37 (17.1; 12.0-22.1)	0.77 (0.45-1.32); 0.346; 0.692; 0.51 (0.49-0.53)	9 (15.3; 6.1-24.4)	10 (15.9; 6.8-24.9)	0.95 (0.36-2.25); 0.925; 0.963; 0.52 (0.48-0.56)
rs61151079 CACGAGG/CACGAGG	2 (1.0; 0.4-2.3)	3 (1.4; 0.2-2.9)	0.71 (0.12-4.27); 0.704; 0.765; 0.51 (0.51-0.52)	0 (0.0; 0.0-0.0)	1 (1.6; -1.5-4.7)	0.00* (0.00-1.97); 0.333; 0.963; 0.51 (0.51-0.52)
rs79792089 G/G	201 (98.5; 96.9-100.2)	213 (98.2; 96.4-99.9)	1.26 (0.28-5.69); 0.765; 0.765; 0.57 (0.20-0.88)	58 (98.3; 95.0-101.6)	62 (98.4; 95.3-101.5)	0.94 (0.06-15.30); 0.963; 0.963; 0.50 (0.03-0.97)
rs79792089 G/A	3 (1.5; 0.2-3.1)	4 (1.8; 0.1-3.6)	0.80 (0.18-3.56); 0.765; 0.765; 0.51 (0.51-0.52)	1 (1.7; -1.6-5.0)	1 (1.6; -1.5-4.7)	1.07 (0.07-17.49); 0.963; 0.963; 0.52 (0.51-0.53)
rs79792089 A/A	0 (0.0; 0.0-0.0)	0 (0.0; 0.0-0.0)	--	0 (0.0; 0.0-0.0)	0 (0.0; 0.0-0.0)	--
**ALLELES**						
rs10947690 A	336 (82.4; 78.7-86.1)	341 (78.6; 74.7-82.4)	1.27 (0.90-1.79); 0.167; 0.440; 0.56 (0.49-0.63)	97 (82.2; 75.3-89.1)	98 (77.8; 70.5-85.0)	1.32 (0.70-2.48); 0.389; 0.897; 0.57 (0.44-0.70)

rs10947690 G	72 (17.6; 13.9-21.3)	93 (21.4; 17.6-25.3)	0.79 (0.56-1.11); 0.167; 0.440; 0.50 (0.49-0.52)	21 (17.8; 10.9-24.7)	28 (22.2; 15.0-29.5)	0.76 (0.40-1.43); 0.389; 0.897; 0.50 (0.47-0.54)
rs61151079 C	376 (92.2; 89.5-94.8)	391 (90.1; 87.3-92.9)	1.29 (0.80-2.09); 0.293; 0.440	109 (92.4; 87.6-97.2)	114 (90.5; 85.4-95.6)	1.28 (0.52-3.15); 0.598; 0.897; 0.57 (0.35-0.77)
rs61151079 CACGAGG	32 (7.8; 5.2-10.5)	43 (9.9; 7.1-12.7)	0.77 (0.48-1.25); 0.293; 0.440; 0.51 (0.50-0.52)	9 (7.6; 2.8-12.4)	12 (9.5; 4.4-14.6)	0.78 (0.32-1.94); 0.598; 0.897; 0.51 (0.49-0.53)
rs79792089 G	405 (99.3; 98.4-100.1)	430 (99.1; 98.2-100.0)	1.26 (0.28-5.65); 0.766; 0.766; 0.57 (0.20-0.88)	117 (99.2; 97.5-100.8)	125 (99.2; 97.7-100.8)	0.94 (0.06-15.14); 0.963; 0.963; 0.50 (0.03-0.97)
rs79792089 A	3 (0.7; 0.1-1.6)	4 (0.9; 0.0-1.8)	0.80 (0.18-3.58); 0.766; 0.766; 0.52 (0.51-0.52)	1 (0.8; 0.8-2.5)	1 (0.8; -0.8-2.3)	1.07 (0.07-17.28); 0.963; 0.963; 0.52 (0.51-0.52)

*** The relative risk is shown instead of the odds ratio because one group in the comparison has a value equal to 0.**

Supplementary Table S2. Genotypes and allelic variants of patients with RLS distributed by response to dopamine agonists, clonazepam and gabaergic drugs. The values in each cell resent number (percentage; 95% confidence intervals). P: crude probability; Pc: probability after multiple comparisons; NPV: negative predictive value.

GENOTYPE	POSITIVE RESPONSE TO DOPAMINE AGONISTS (N=205, 410 ALLELES)	NEGATIVE RESPONSE TO DOPAMINE AGONISTS (N=16, 32 ALLELES)	INTERGROUP COMPARISON VALUES *OR (95%CI) P ; PC ; NPV OR (95%CI)*	POSITIVE RESPONSE TO CNZ (N=89, 178 ALLELES)	NEGATIVE RESPONSE TO CNZ (N=16, 32 ALLELES)	INTERGROUP COMPARISON VALUES *OR (95%CI) P ; PC ; NPV OR (95%CI)*	POSITIVE RESPONSE TO GABAERGIC DRUGS (N=49, 98 ALLELES)	NEGATIVE RESPONSE TO GABAERGIC DRUGS (N=11, 22 ALLELES)	INTERGROUP COMPARISON VALUES *OR (95%CI), P ; PC ; NPV OR (95%CI)*
rs10947690 A/A	134 (65.4; 58.9-71.9)	12 (75.0; 53.8-96.2)	0.63 (0.20-2.02); 0.434; 0.615; 0.05 (0.02-0.11)	58 (65.2; 55.3-75.1)	10 (62.5; 38.8-86.2)	1.12 (0.37-3.38); 0.838; 0.838; 0.16 (0.07-0.27)	36 (73.5; 61.1-85.8)	6 (54.5; 25.1-84.0)	2.31 (0.60-8.86); 0.220; 0.770; 0.28; (0.12-0.45)
rs10947690 A/G	66 (32.2; 25.8-38.6)	4 (25.0; 3.8-46.2)	1.42 (0.44-4.59); 0.522; 0.615; 0.08 (0.05-0.10)	26 (29.2; 19.8-38.7)	6 (37.5; 13.8-61.2)	0.69 (0.23-2.09); 0.509; 0.729; 0.14 (0.08-0.18)	12 (24.5; 12.4-36.5)	5 (45.5; 16.0-74.9)	0.39 (0.10-1.51); 0.167; 0.770; 0.14 (0.07-0.21)
rs10947690 G/G	5 (2.4; 0.3-4.6)	0 (0.0; 0.0-0.0)	1.08* (0.51-1.08); 0.528; 0.615; 0.07 (0.06-0.07)	5 (5.6; 0.8-10.4)	0 (0.0; 0.0-0.0)	1.19* (0.56-1.19); 0.334; 0.729; 0.16 (0.13-0.16)	1 (2.0; -1.9-6.0)	0 (0.0; 0.0-0.0)	1.23* (0.07-1.23); 0.636; 0.882; 0.19 (0.17-0.19)
rs61151079 C/C	176 (85.9; 81.1-90.6)	13 (81.3; 62.1-100.4)	1.40 (0.38-5.22); 0.615; 0.615; 0.09 (0.03-0.22)	79 (88.8; 82.2-95.3)	12 (75.0; 53.8-96.2)	2.63 (0.71-9.75); 0.138; 0.552; 0.29 (0.10-0.54)	41 (83.7; 73.3-94.0)	9 (81.8; 59.0-104.6)	1.13 (0.21-6.29); 0.882; 0.882; 0.20 (0.04-0.51)
rs61151079 C/CACGAGG	27 (13.2; 8.5-17.8)	3 (18.8; -0.4-37.9)	0.66 (0.18-2.46); 0.531; 0.615; 0.07 (0.05-0.08)	9 (10.1; 3.8-16.4)	4 (25.0; 3.8-46.2)	0.34 (0.09-1.27); 0.098; 0.552; 0.13 (0.09-0.16)	8 (16.3; 6.0-26.7)	2 (18.2; -4.6-41.0)	0.88 (0.16-4.85); 0.882; 0.882; 0.18 (0.12-0.21)
rs61151079 CACGAGG/CACGAGG	2 (1.0; 0.4-2.3)	0 (0.0; 0.0-0.0)	0.97* (0.80-1.06); 0.531; 0.615; 0.07 (0.05-0.08)	1 (1.1; -1.1-3.3)	0 (0.0; 0.0-0.0)	1.18* (0.06-1.18); 0.672; 0.768; 0.15 (0.15-0.15)	0 (0.0; 0.0-0.0)	0 (0.0; 0.0-0.0)	--
rs79792089 G/G	201 (98.0; 96.2-99.9)	16 (100.0; 100.0-100.0)	0.93* (0.93-2.28); 0.574;	87 (97.8; 94.7-100.8)	16 (100.0; 100.0-	0.85* (0.85-4.27); 0.547;	48 (98.0; 94.0-101.9)	11 (100.0; 100.0-100.0)	0.81* (0.81-15.11); 0.636; 0.882; 0.00

			0.615; 0.00 (0.00-0.59)		100.0)	0.729; 0.00 (0.00-0.80)			(0.00-0.95)
rs79792089 G/A	4 (2.0; 0.1-3.8)	0 (0.0; 0.0-0.0)	1.08* (0.44-1.08); 0.574; 0.615; 0.07 (0.06-0.07)	2 (2.2; -0.8-5.3)	0 (0.0; 0.0-0.0)	1.18* (0.23-1.18); 0.547; 0.729; 0.16 (0.14-0.16)	1 (2.0; -1.9-6.0)	0 (0.0; 0.0-0.0)	1.23* (0.07-1.23); 0.636; 0.882; 0.19 (0.17-0.19)
rs79792089 A/A	0 (0.0; 0.0-0.0)	0 (0.0; 0.0-0.0)	--	0 (0.0; 0.0-0.0)	0 (0.0; 0.0-0.0)	--	0 (0.0; 0.0-0.0)	0 (0.0; 0.0-0.0)	--
ALLELES									
rs10947690 A	334 (81.5; 77.7-85.2)	28 (87.5; 76.0-99.0)	0.63 (0.21-1.84); 0.394; 0.711; 0.05 (0.02-0.12)	142 (79.8; 73.9-85.7)	26 (81.3; 67.7-94.8)	0.91 (0.35-2.38); 0.848; 0.848; 0.14 (0.06-0.27)	84 (85.7; 78.8-92.6)	17 (77.3; 59.8-94.8)	1.77 (0.56-5.55); 0.329; 0.887; 0.26 (0.11-0.48)
rs10947690 G	76 (18.5; 14.8-22.3)	4 (12.5; 1.0-24.0)	1.59 (0.54-4.68); 0.394; 0.711; 0.08 (0.06-0.09)	36 (20.2; 14.3-26.1)	6 (18.8; 5.2-32.3)	1.10 (0.42-2.87); 0.848; 0.848; 0.16 (0.12-0.18)	14 (14.3; 7.4-21.2)	5 (22.7; 5.2-40.2)	0.57 (0.18-1.78); 0.329; 0.887; 0.17 (0.13-0.20)
rs61151079 C	379 (92.4; 89.9-95.0)	29 (90.6; 80.5-100.7)	1.27 (0.37-4.39); 0.711; 0.711; 0.09 (0.02-0.23)	167 (93.8; 90.3-97.4)	28 (87.5; 76.0-99.0)	2.16 (0.65-7.29); 0.202; 0.202; 0.27 (0.09-0.53)	90 (91.8; 86.4-97.3)	20 (90.9; 78.9-102.9)	1.13 (0.22-5.71); 0.887; 0.887; 0.20 (0.04-0.54)
rs61151079 CACGAGG	31 (7.6; 5.0-10.1)	3 (9.4; -0.7-19.5)	0.79 (0.23-2.74); 0.711; 0.711; 0.07 (0.06-0.08)	11 (6.2; 2.6-9.7)	4 (12.5; 1.0-24.0)	0.46 (0.14-1.55); 0.202; 0.202; 0.14 (0.12-0.16)	8 (8.2; 2.7-13.6)	2 (9.1; -2.9-21.1)	0.89 (0.18-4.51); 0.887; 0.887; 0.18 (0.15-0.20)
rs79792089 G	406 (99.0; 98.1-100.0)	32 (100.0; 100.0-100.0)	0.93* (0.93-2.32); 0.575; 0.711; 0.00 (0.00-0.60)	176 (98.9; 97.3-100.4)	32 (100.0; 100.0-100.0)	0.85* (0.85-4.28); 0.548; 0.548; 0.00 (0.00-0.80)	97 (99.0; 97.0-101.0)	22 (100.0; 100.0-100.0)	0.82* (0.82-15.03); 0.636; 0.887; 0.00 (0.00-0.95)
rs79792089 A	4 (1.0; 0.0-1.9)	0 (0.0; 0.0-0.0)	1.08* (0.43-1.08); 0.575; 0.711; 0.07 (0.07-0.07)	2 (1.1; -0.4-2.7)	0 (0.0; 0.0-0.0)	1.18* (0.23-1.18); 0.548; 0.548; 0.15 (0.15-0.15)	1 (1.0; -1.0-3.0)	0 (0.0; 0.0-0.0)	1.23* (0.07-1.23); 0.636; 0.887; 0.19 (0.18-0.19)

*** The relative risk is shown instead of the odds ratio because one group in the comparison has a value equal to 0.**

Supplementary Table S3. Cross-population comparison of SNV frequencies using data from gnomAD

SNV	European (non Finnish). Includes Spaniards	East Asians	South Asians	Africans
rs10947690	0.2554	0.0056	0.1244	0.0387
rs61151079	0.0997	0.2224	0.1776	0.2287
rs79792089	0.0122	0.0006	0.0410	0.0002
Common missense SNVs	0.3673	0.2286	0.3430	0.2676

Supplementary Table S4. Calculation of statistical power for *MDGA1* rs10947690, rs61151079, and rs79792089 variants. Alpha = 5%.

SNV	MAF RLS patients (526 alleles)	MAF controls (560 alleles)	OR 1.5, one tailed	OR 1.5, two tailed	OR 1.75 one tailed	OR 1.75 one tailed	OR 4.0 one tailed	OR 4.0 two tailed
rs10947690 G	0.177	0.216	89.40%	82.44 %	99.22%	98.23%	100 %	100 %
rs61151079 CACGAGG	0.078	0.098	69.05%	57.23%	91.80%	85.91%	100 %	100 %
rs79792089 A	0.008	0.009	17.15%	10.34 %	25.76%	16.74 %	89.93%	83.10%
